# Criteria and treatment decisions in the management of deep caries lesions: Is there endodontic overtreatment?

**DOI:** 10.4317/jced.55050

**Published:** 2018-08-01

**Authors:** Isabel Crespo-Gallardo, Olesia Hay-Levytska, Jenifer Martín-González, Mari-Carmen Jiménez-Sánchez, Benito Sánchez-Domínguez, Juan J. Segura-Egea

**Affiliations:** 1 DDS, Department of Stomatology – Endodontic Section, School of Dentistry, University of Sevilla, C/ Avicena S/N, 41009 Sevilla, Spain; 2 DDS, PhD, Department of Stomatology – Endodontic Section, School of Dentistry, University of Sevilla, C/ Avicena S/N, 41009 Sevilla, Spain; 3 DDS, MSc, Department of Stomatology – Endodontic Section, School of Dentistry, University of Sevilla, C/ Avicena S/N, 41009 Sevilla, Spain; 4 MD, DDS, PhD, Department of Stomatology – Endodontic Section, School of Dentistry, University of Sevilla, C/ Avicena S/N, 41009 Sevilla, Spain

## Abstract

**Background:**

The aim of this study was to investigate the diagnostic criteria and treatment decisions in the management of deep caries lesions (DCLs). The null hypothesis tested was that DCLs are managed according to the current scientific evidence.

**Material and Methods:**

A total of 288 dentists were contacted directly or by mail, and 125 (43%) were included in the study. Dentists were requested to answer a questionnaire about the routine approach to the diagnosis and treatment of DCLs. Logistic regression analyses were carried out to calculate odds ratios (OR).

**Results:**

Pulp sensitivity tests were used by 65% of dentists when assessing pulpal health in cases of DCLs, particularly those who had followed courses in cariology (OR = 3.8; *p* = 0.005). Dentine hardness was the most frequent criterion used during DCLs excavation (98%). Two thirds of the respondents (65%) removed carious tissue until they felt hard dentine, and feeling hard dentine correlated with caries removal even at the risk of pulpal exposure (OR = 15.8; *p* = 0.0000). Acute transient pain or sensitivity to cold or heat (reversible pulpitis) were considered by 58% of respondents as a reason to provide endodontic therapy.

**Conclusions:**

The null hypothesis tested is rejected. The evidence-based more conservative approach on the management of DCLs is not being translated to clinical dentistry. Root canal treatment is being indicated in cases of DCLs in which the diagnosis is reversible pulpitis. Likewise, it can be concluded that non-conservative management of DCLs, with endodontic overtreatment, could be occurring.

** Key words:**Deep caries lesions, dental pulp capping, dental pulp health, dentists, endodontic therapy, pulpal diagnosis, reversible pulpitis, treatment decisions.

## Introduction

The caries lesion is the localized destruction of the susceptible dental hard tissues caused by acids formed by the oral bacteria as final products of the fermentation of the diet carbohydrates (1,2). Since the origin of the dental profession, the treatment of carious lesions has been a main part of the dental clinical practice. Dentists should decide when, how and to what extent to remove carious tissue before the placement of a restoration, considering the restorability of the tooth, preservation of tooth structure and pulp vitality (3). The European Society of Endodontology (4) recommended the appropriate early treatment of carious lesions, keeping the cavity preparations as small as possible, to contribute to the maintenance of pulp health. The treatment procedures for reversible pulp damage are indirect (stepwise excavation) and direct pulp capping. ESE indicates root canal treatment only when there is irreversible pulp damage (4).

Diagnosing pulp status is the key to the treatment decision. However, both the diagnostic criteria to assess pulp status and the treatment decisions applied by each dentist in the management of deep caries lesions (DCLs) are highly variable (5,6). The variability of criteria is especially evident in relation to the depth of the excavation of the carious tissue, ranging from the complete and non-selective removal of carious tissue to hard dentin, with the risk of pulp exposure and endodontic treatment, to the selective excavation that leaves soft and affected dentine in the central area of the lesion near the pulp (5,7).

The International Caries Consensus Collaboration Group (ICCC), linked to the International Association for Dental Research - Cariology Group, has established well-defined criteria for the treatment of DCLs (8,9). The complete excavation or removal of carious dentine is currently considered over-treatment (6,8,9,10) but several surveys carried out in different countries indicate that many dentists continue this practice (5,11-14). Moreover, some dental schools still recommended the elimination of the bacteria present in soft dentine, forgetting other ways to fight the infection (5,7).

Especially worrying are the results obtained in surveys conducted in USA (5), Brazil (11) and central and northern Europe (12-14), showing disturbing results regarding the therapeutic decision making in cases of DCLs in which the pulpal diagnosis was reversible pulpitis. However, no study has investigated this topic in southern Europe. The aim of this study was to conduct a survey amongst dentists to investigate the diagnostic criteria and treatment decisions in the management of DCLs. The null hypothesis tested was that DCLs are managed according to the knowledge and principles derived from current scientific evidence and ESE recommendations.

## Material and Methods

The present study was carried out in the south of Europe (Sevilla, Andalucía, Spain) during October 2017 to February 2018. The survey included dentists working or attending postgraduate courses in the Dental School of the University of Sevilla, with both private and public clinical practice. A total of 288 dentists were contacted directly or by mail, and 134 (46.5%) fulfilled the survey, being excluded 6 dentists because they answered the questionnaire incompletely and 3 dentists because they were no longer practicing clinical activities. Therefore, 125 (43.4%) dentists were included in the study. The purpose of the study was explained to all and indicated confidential and anonymous processing of the data. Ethical approval of this study was considered unnecessary by the Ethical Committee of de University of Sevilla.

Respondent dentists were requested to answer an open/discursive questionnaire ( Table 1,  Table 1 continue) based in previous surveys carried out in USA (5), Brazil (11), and Europe (12-14). Translation of the English master versions was performed by native speakers. Briefly, after several questions concerning the respondents’ demographic, educational, and professional backgrounds, the item batteries comprised: 1) habits for routinely approach the diagnosis of DCLs, 2) criteria to assess carious tissue removal, 3) methods for carious dentine removal when near the pulp, 4) routine habits to approach and treatment decisions in the management of DCLs, and 5) liner or base materials used for different indications.

**Table 1:**
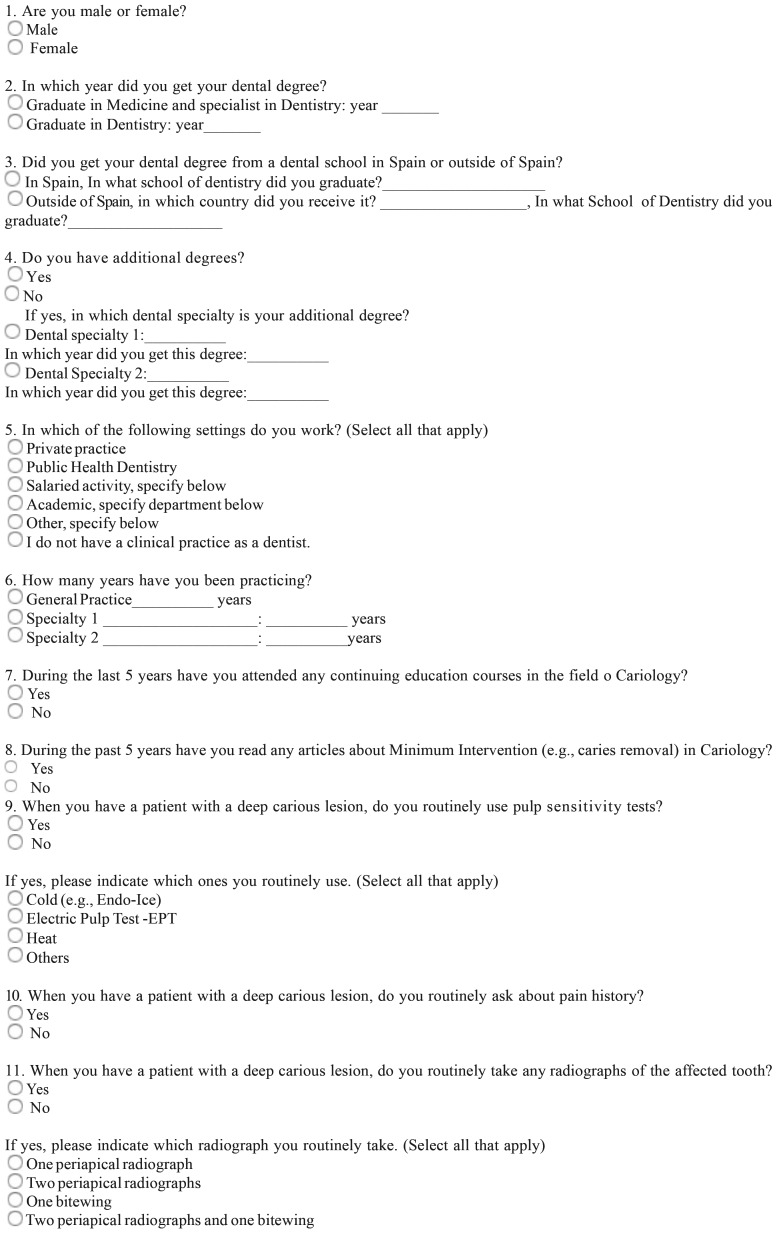
Questionnaire.

**Table 1 continue:**
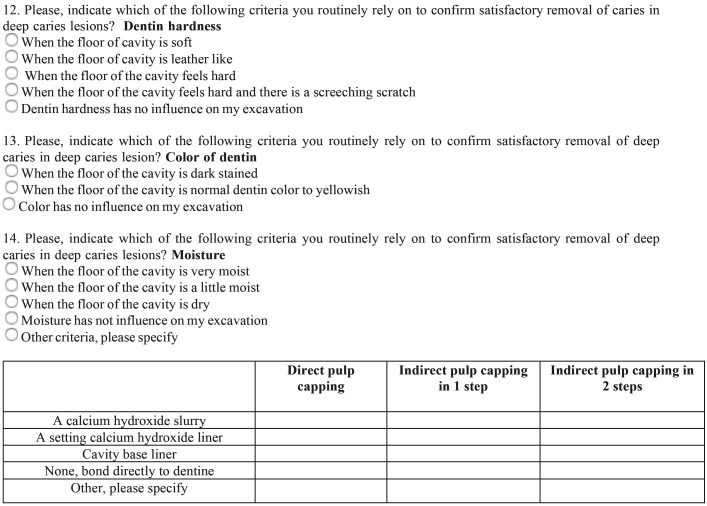
Questionnaire.

-Statistical analysis

A database was created for further analysis using Excel (Microsoft Corp., Redmond, WA, USA). Data description was carried out by frequency tables to provide an overview of the responses. When obtaining the numerical representation by percentages, the total number of answers for each query was taken into account. Normal distribution of data was controlled using the Shapiro-Wilk test. Logistic regression analysis was carried out transforming qualitative explanatory variables into binary variables. Odds ratios (OR) and confidence intervals (CI) were calculated as effect estimates. Significant differences were considered when *p* < 0.05.

## Results

-Respondents

Among the 125 dentists respondents to the survey, 27.2% were male and 72.8% were female ( Table 2). Most of the respondents received their dental education in Spain (119; 95.2%), and 73 (58.4%) received specialized training in some dental specialty (14.4% periodontics-implantology, 12% endodontics, 8.8% oral surgery, 8.8% prosthodontics and 8% orthodontics). The mean year of graduation from dental school was 2010 (range, 1983-2017), ranging the number of years in practice from 0.5 to 35 years (mean, 7.1 years). The vast majority of respondents worked in private practice (93; 74.4%), and 19.2% worked in academia, teaching dentistry at the University. Fifty seven (45.6%) respondents attended a continuing education course about cariology in the past 5 years, and ninety seven (77.6%) read articles about minimal intervention in the treatment of caries lesions.

**Table 2:**
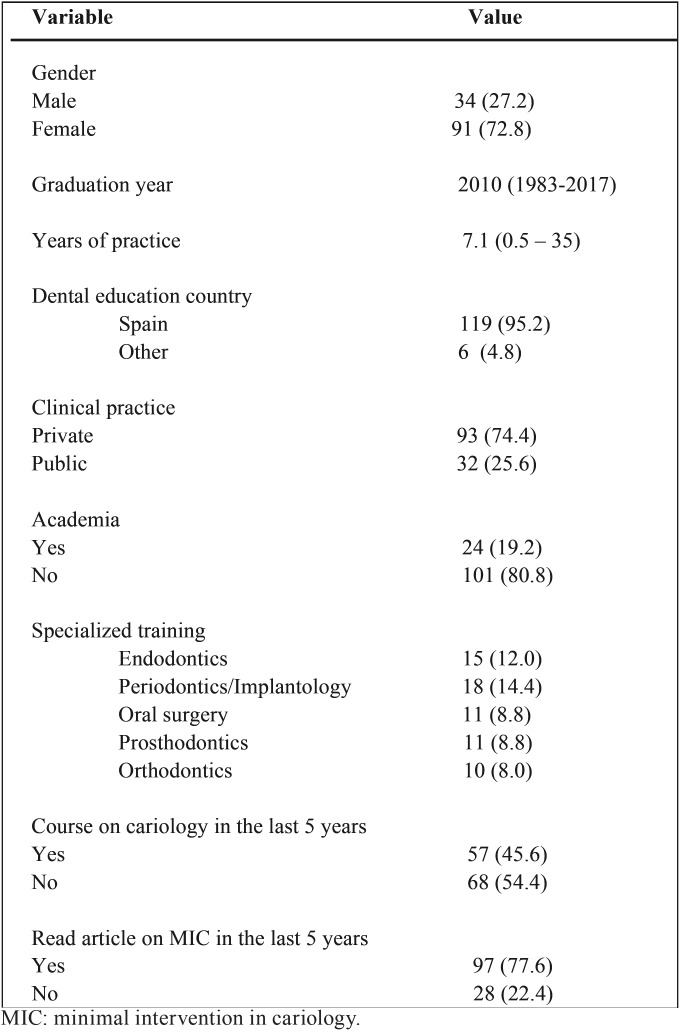
Demographic, academic and professional variables.

Criteria for routinely approach the diagnosis of DCLs and carious tissue removal

The second part of the survey asked about the criteria for routinely approach the diagnosis of deep caries lesions ( Table 3). More than a half of the respondents (60%) indicated that they routinely used some pulp sensitivity test, but more than a third (40%) did not use any. Cold tests (59.2%) and heat tests (12%) were the most used pulp sensitivity tests. There were no statistically significant differences among dentists in the use of pulp tests according to the type of specialized training they received (*p* > 0.05). However, the use of pulp test was significantly more frequent by dentists who attended course on cariology in the past 5 years (OR = 3.8; 95% C.I. = 1.5 – 9.9; *p* = 0.005). Almost all dentists (96.0%) asked about their patients’ pain history and obtained radiographs of a tooth with a carious lesion (93.6%), but only 5.6% routinely obtained 2 periapical radiographs, and 11.2% one bitewing radiograph for their diagnosis.

**Table 3:**
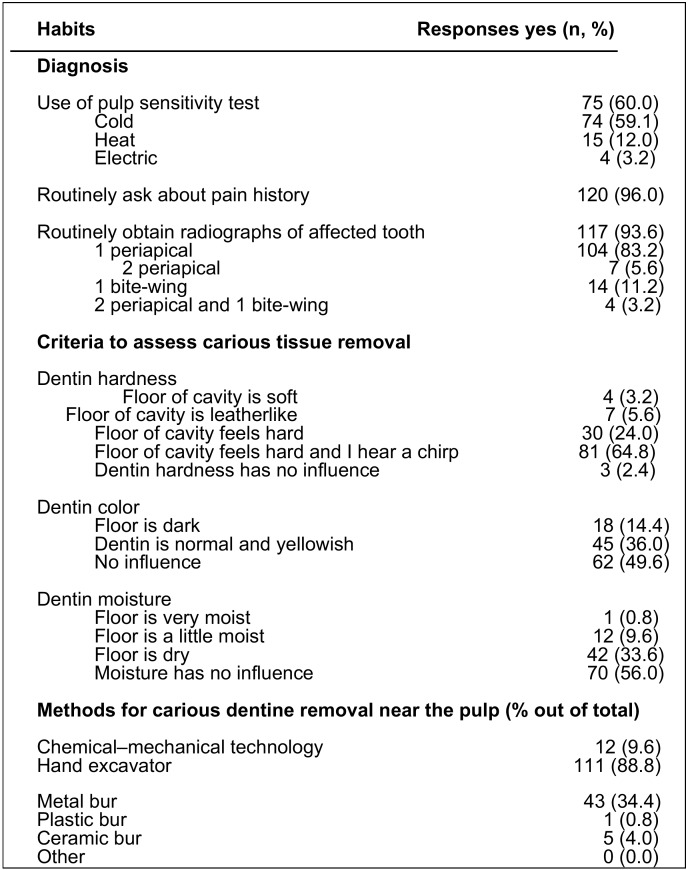
Routinely approach to diagnosis of deep carious lesions and carious tissue removal.

They were also asked about the criteria followed to confirm that the carious tissue had been completely removed ( Table 3). The hardness of the floor of the cavity was the criterion most frequently (98%) taken into account by the respondents to confirm the satisfactory removal of the carious tissue. Most of the dentists (65%) used the criterion that the floor of the cavity feels hard and, in addition, it heard the scratching of the hand excavator. Only 3.2% of respondents chose the criterion of soft dentine. Regarding the dentine color, almost half of the respondents (49.6%) did not value the color of the dentine during carious tissue removal. More than half of the dentists (65.0%) did not value the moisture of the dentine. Hand excavators (88.8%) and metal burs (34.4%) were the most-used instruments to remove the dentine near the pulp.

-Treatment decisions in the management of DCLs

Regarding the routine approach for treating deep carious lesions ( Table 4), less than half of the dentists used rubber dams (42.4%) during the treatment of deep carious lesions, and approximately one-half used caries indicator (44.8%) and/or antibacterials under restorations (46.4%). Routine follow-up visits after the treatment were used by 73.6% of respondents. Most of the respondents (57.6%) considered symptoms (acute transient pain or sensitivity to cold or heat) as a reason to provide endodontic therapy. To the question “what treatment would you perform when during the excavation of a deep carious lesion, in an asymptomatic patient, a pulpal exposure occurs”, the majority (79.2%) chose to perform direct pulp capping, but 20.8% would carry out root canal treatment. On the contrary, in the case of pulpal exposure in a patient with symptoms, most (83.2%) would prefer to perform endodontic therapy.

**Table 4:**
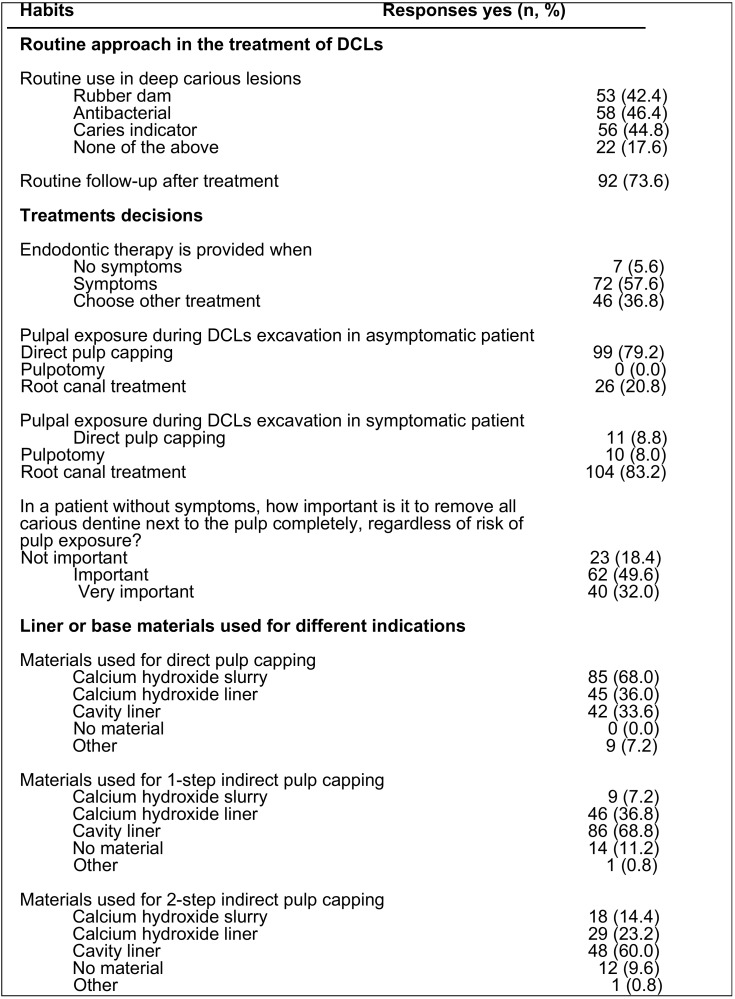
Routine approach and treatment decisions in the management of deep carious lesions (DCLs).

To the question about how important is it, in an asymptomatic patient, to completely remove all carious dentine next to the pulp, with risk of pulp exposure, most of dentists thought it was important (49.2%) or very important (32.3%). Logistic regression demonstrated significant correlation between respondents who considered important to attain complete caries excavation, even at risk of pulp exposure, and dentists who used to get to hard dentin as criteria for caries removal (OR = 15.8; 95% C.I. = 4.3 – 58.1; *p* = 0.0000).

When were asked the type of materials used for direct and indirect pulp capping ( Table 4), more than two thirds of dentists (68.0%) used calcium hydroxide slurry, and 36% calcium hydroxide liner in direct pulp capping. In the case of one-step indirect pulp capping, cavity baseliner was the preferred material (68.8%), followed by calcium hydroxide liner (36.8%). Finally, for indirect pulp capping in 2 steps, cavity base liner (48.0%) and calcium hydroxide liner (23.2%) were also the preferred materials.

## Discussion

This study aimed to investigate whether the new approaches to decision-making and surgical therapy of DCLs, derived from current scientific evidence and ESE recommendations (4), are being incorporated into the diagnostic criteria and treatment decisions of dental practitioners. The questions included in the study were based on previously published surveys (5,11,12,13), whose questions related to the follow-up by dentists of diagnostic and therapeutic criteria based on current scientific evidence in relation to the management of DCLs.

After the joint assessment of the answers given by respondents, the main result of the present study is that new concepts about the more conservative approach to DCLs based in the current scientific evidence, have not yet been completely translated to clinical dentistry. The null hypothesis tested is rejected. Root canal treatment is being indicated in cases of DCLs in which the diagnosis is reversible pulpitis and vital pulp therapy is indicated. Likewise, it can be concluded that endodontic overtreatment is occurring.

The population sampled was Spanish dentists from Andalusia (Spain, southern Europe), and the sample size and the overall response rate were similar to other published surveys conducted under equivalent conditions (5,12,13). The majority of respondents were women (73%), reflecting the feminization of the dental profession in Spain and other countries, already evident in previous studies (11,15,16).

Diagnosing pulp status from clinical examination and patient’s symptoms is a challenge in clinical practice (17), being pulpal diagnosis the key to make therapeutic decisions in cases of DCLs. The treatment procedures for reversible pulp damage are indirect or direct pulp capping, and RCT only is indicated in cases of irreversible pulpitis or necrotic pulp (4). The patient’s history of pain, experience of trauma or restorative procedures, clinical and radiographic examination results, and clinical test results can give enough information to decide the treatment to be performed (17). Pulpal sensibility testing with cold combined with an electric pulp tester are accurate and reliable methods of determining pulpal vitality (92% sensitivity and 90% specificity) (18). However, when the relationship between patient complaints and histopathologic diagnosis of pulpal condition have been analyzed, results have shown that finding that pain on cold stimuli was present in 100% of patients with untreatable pulpal states (100% sensitivity) but also was present in 71% of patients with treatable pulpal states (28.6% specificity) (19). Although the pulp response to the cold does not differentiate accurately if an irreversible pulpitis already exists, in regular dental practice the cold test has validity to discriminate between vital and nonvital pulp (20).

In the present study, almost all dentists (96.0%) asked about their patients’ pain history, in accordance with previous surveys (5). However, only 60% of respondents used routinely some pulp sensitivity test, a higher percentage than that found in USA (44%) by Koopaeei *et al.* (5). Cold tests (59%) was the most used, in agreement with the results of the survey carried out in USA (39%) (5). It should be noted the significant correlation between having received courses on cariology and the use of pulp sensitivity tests (OR = 3.8; *p* = 0.005), which highlights that continuing training in cariology is very important to motivate and update the dental practitioner.

Radiographs provide essential information about the presence and depth of caries lesion and, when different angulations are used, pulpal involvement can be accurately assessed (21). Dental practitioner should assess the risks and benefits of the use of radiographs in each individual case, according to the ALARA principle, especially in children, minimizing the prescription of radiographs (5). In this study, 94% of respondents used radiographs, most one periapical radiograph (83%), in their examination and diagnosis process. Although these percentages are in accordance with the results of other surveys, only 11% of respondents used bite-wing radiographs, a percentage considerably lower than that found by Koopaeei *et al.* (5) in USA (44%). Taking into account that bite-wing radiographs are the most recommended adjunct method diagnosing the location and size of DCLs in clinically inaccessible proximal surfaces (22,23). Dentists could be underestimating the incidence and extension of DCLs in these surfaces.

Dentine hardness is the criterion recommended by the International Caries Consensus Collaboration (ICCC) group to determine the clinical consequences of the disease and to define how far should go the removal of carious dentine (8). Accordingly, the hardness of the floor of the cavity was the most frequently criterion (98%) used by respondents to confirm the satisfactory removal of the carious tissue, in agreement with the results of previous studies in USA and Europe (5,12,14).

On the contrary, although researches carried out in the two last decades have shown that bacterially contaminated or demineralized tissues close to the pulp (soft dentine) do not need to be removed (6,8,10,24,25), almost two thirds of the dentists (65%) removed carious tissue until they felt hard dentine. Unfortunately, the same, or worse, results have been found in surveys conducted in USA (5), Germany (12), France and Norway (13,14). The concept that carious dentine that is adequately sealed will remineralize, resulting in great hardness and stiffness (26,27), seems not to be assumed by dentists who, for many years, have routinely removed the carious tissue to the hard dentine. While the ICCC group (8) considered that moisture and color of dentine are not good references to determine the amount of tissue that should be removed, 50% and 44% of respondents valued the color or the moisture of the dentine, respectively. These results are also in accordance with those of previous surveys (5,14).

Regarding dentine removal when near the pulp, most of the respondents (89%) preferred hand excavators, a higher percentage than that (62%) found in the previous study of Koopaeei *et al.* (5), in which 74% of dentists preferred to remove the carious tissue with metal burs. On the contrary, the survey conducted by Schwendicke *et al.* (14) revealed that German, French and Norwegian dentists preferred metal burs to hand excavator to remove the dentine near the pulp. Hand or chemomechanical excavation might reduce pain and discomfort during treatment, although there is insufficient evidence to recommend any single method for carious tissue removal (8).

About the use of dye solutions, although stainability via caries detector dyes lack sufficient clinical validation (28) almost half of dentists (45%) used the staining with caries indicator to assess carious tissue removal. This percentage is higher than those found in other European countries (14) and in USA (5). Less than half (43%) of the dentists reported using rubber dam during the treatment of DCLs, a percentage similar to that found in German general dentists (48%) (14), but greater than those found in general dentists in USA (31%) (8), France (18%) and Norway (13%) (14). All these percentages are very low compared to that of endodontists (5,29). According to the ESE and the AAE, using a rubber dam is the standard of care during all endodontic procedures (4,30). Regarding cavity disinfection, 46% of respondents used antibacterials under restorations, a result similar to that found in general dentists of USA (48%) (12), but lower than those found in French (74%) and German (74%) dentists (14). The use of antibacterials to disinfect the cavity is not supported by scientific evidence and can unnecessarily increase the time and cost of treatment (8).

The ESE recommended clinical and radiographic follow-ups at regular intervals for a minimum observation period of 1 year (4). Seventy four percent of respondents used routine follow-up visits after the treatment, a high percentage compared to that of dentists in USA (52%) (8). Dentists should encourage patients to return at appropriate follow-up intervals for evaluation (30).

Regarding pulp capping, after washing and drying, the cavity must be covered with material(s) that protect(s) the pulp from additional injury and permit(s) healing and repair (4). In the present study, most of dentists used calcium hydroxide, slurry (68%) or liner (36%), in direct pulp capping. Similar results have been found in previous surveys (5). However, 8% preferred use other materials than the ones listed in the survey, such as new biocompatible bioceramic materials. Similarly, 93% of American endodontists preferred “other materials” than calcium hydroxide for direct pulp capping (5). For indirect pulp capping (both 1 step and 2 steps) cavity baseliner was the most common material, coinciding with the results found in the survey carried out in USA (5). Although cavity lining has been used in treating DCLs to reduce the number of residual viable bacteria, remineralize dentine, induce reactionary dentine, isolate the pulp, and protect pulpal cells from noxious stimuli (31), the scientific evidence supporting its use is sparse and its clinical relevance unclear (8). However, cavity-lining materials might impeding monomer penetration and avoidance of fracture of the remaining dentine in composite resin restorations (8,32,33).

When the diagnosis of a tooth with DCL is reversible pulpitis (patient without spontaneous pain and no lingering pain to cold test following the removal of the stimulus) and there is not pulp exposure, the priority is to maintain pulpal health (4,30). However, in the present study, more than half of the dentists (58%) considered the presence of sharp transient pain or sensitivity to cold or heat as a reason to provide endodontic therapy. This percentage indicates a high degree of endodontic overtreatment, but it is lower than that found in the survey developed in the USA (82%) (5). On the contrary, only 39% of Norwegian dentists preferred root canal treatment in this clinical scenario (13).

Direct pulp capping is the preferred treatment options when pulp exposure occurs during caries excavation in a tooth without symptoms (reversible pulpitis) (30). This procedure may be performed when the pulp is exposed through noninfected dentine and the tooth has no recent history of spontaneous pain and a bacteria-tight seal can be applied (4). But if bleeding cannot be controlled, diagnosis changes to irreversible pulpitis, being indicated RCT (4,30). In the present study, in case of pulp exposure during DCLs excavation in asymptomatic patient, 79% of respondents preferred vital pulp therapy and only 21% indicated RCT, the same percentage (21%) found by Oen *et al.* (34). In the studies of Stangvaltaite *et al.* (13) and Koopaeei *et al.* (5), 42% y 79% of dentists, respectively, chose to perform endodontic therapy in cases of pulp exposure in asymptomatic patients. A tooth is considered symptomatic when subjective symptoms of sharp transient pain to cold or hot are presents, indicating a more severe pulpal inflammation (13). Even if pain is an uncertain diagnostic criterion (35), pronounced and persistent toothache is the key symptom of irreversible pulpitis. In the case of pulpal exposure in a symptomatic patient, most (83%) of respondents preferred to perform RCT. Similar results were obtained in the surveys conducted in USA (79%) (5) and in Norway (91%) (13).

Regarding the knowledge and factors underlying their excavation strategy, most of the respondents (81.5%) considered important or very important completely remove all carious dentine next to the pulp in the treatment of a DCL in an asymptomatic patient, although there is risk of pulp exposure. In the answer to this question, a very strong and significant correlation was observed between those who used hard dentine as criterion for caries removal and those who completely eliminated the dentine near the pulp (OR = 15.8; *p* = 0.0000). This finding is in accordance with the correlation between the preference for an excavation strategy and the perceived danger or benefits of sealing carious lesions found by Schwendicke *et al.* (14) in their survey amongst European dentists. Similar results were reported in USA by Koopaeei *et al.* (5), who found that 84% of dentists considered important or very important to attain complete caries removal even at risk of pulp exposure.

The difficulty of transferring current knowledge about the management of DCLs, derived from evidence-based dentistry, to clinical practice could be due to a lack of update of the Caryology and Endodontic programs. Dental Schools, especially the teaching teams of Caryology and Endodontics, as well as continuing dental education courses, should modify their teaching programs incorporating the current scientific evidence and the clinical guidelines (4,30) supporting the use of conservative criteria for caries removal to preserve pulpal health and the dental structure when managing DCLs.

## Conclusions

After the joint assessment of the answers given by respondents, the main result of the present study is that the more conservative approach on the treatment of DCLs is not being translated by Spanish dentists into daily clinical practice. On the contrary, it seems that endodontic overtreatment could be occurring.
