# Correction: Criteria and treatment decisions in the management of deep caries lesions: Is there endodontic overtreatment?

**DOI:** 10.4317/jced.532742

**Published:** 2019-01-01

**Authors:** Isabel Crespo-Gallardo, Olesia Hay-Levytska, Jenifer Martín-González, Mari-Carmen Jiménez-Sánchez, Benito Sánchez-Domínguez, Daniel Cabanillas-Balsera, Juan J. Segura-Egea

**Affiliations:** 1 DDS, Department of Stomatology – Endodontic Section, School of Dentistry, University of Sevilla, C/ Avicena S/N, 41009 Sevilla, Spain; 2 DDS, PhD, Department of Stomatology – Endodontic Section, School of Dentistry, University of Sevilla, C/ Avicena S/N, 41009 Sevilla, Spain; 3 DDS, MSc, Department of Stomatology – Endodontic Section, School of Dentistry, University of Sevilla, C/ Avicena S/N, 41009 Sevilla, Spain; 4 MD, DDS, PhD, Department of Stomatology – Endodontic Section, School of Dentistry, University of Sevilla, C/ Avicena S/N, 41009 Sevilla, Spain

In this article by Crespo-Gallardo and colleagues ( *J Clin Exp Dent* 2018;8: 751-760 1 Aug), there is an error in the authors of the manuscript. The correct author list is: Isabel Crespo-Gallardo,Olesia Hay-Levytska,Jenifer Martín-González,María del Carmen Jiménez-Sánchez,Benito Sánchez-Domínguez,Daniel Cabanillas-Balsera,Juan J. Segura-Egea.
